# Development of an autoantibody panel for early detection of lung cancer in the Chinese population

**DOI:** 10.3389/fmed.2023.1209747

**Published:** 2023-11-27

**Authors:** Lin Tong, Jiayuan Sun, Xiaoju Zhang, Di Ge, Ying Li, Jian Zhou, Dong Wang, Xin Hu, Hao Liu, Chunxue Bai

**Affiliations:** ^1^Department of Pulmonary and Critical Care Medicine, Zhongshan Hospital, Fudan University, Shanghai, China; ^2^Shanghai Respiratory Research Institute, Shanghai, China; ^3^Department of Respiratory Endoscopy, Shanghai Chest Hospital, Shanghai Jiao Tong University School of Medicine, Shanghai, China; ^4^Department of Respiratory and Critical Care Medicine, Henan Provincial People's Hospital, Zhengzhou, China; ^5^Department of Thoracic Surgery, Zhongshan Hospital, Fudan University, Shanghai, China; ^6^Gene Tech (Shanghai) Company Limited, Shanghai, China

**Keywords:** lung cancer, early detection, autoantibody, pulmonary nodule, Chinese population

## Abstract

**Introduction:**

Tumor-associated autoantibodies have been revealed as promising biomarkers for the early detection of lung cancer. This study was designed to develop an autoantibody panel for early detection of lung cancer in the Chinese population.

**Methods:**

Recruited prospectively in three clinical centers, the subjects (*n* = 991) who had a definite diagnosis during follow-up were included in the development of the autoantibody panel. The levels of 14 autoantibody candidates in plasma were detected.

**Results:**

A panel of nine autoantibody markers (named as CN9), namely, P53, SOX2, SSX1, HuD, NY-ESO-1, CAGE, MAGE-A4, P62, and CK20, was preferably selected from 14 candidates. The overall specificity, sensitivity, and AUC were 90.5%, 40.8%, and 0.64, respectively. The CN9 panel demonstrated a reasonable detection rate in lung cancer patients at all stages, histological types, sizes of lesions, and risk levels. Its estimated overall accuracy is 85.5% and 90%, with PPV at 0.32 and 0.04, and NPV at 0.93 and 0.99 in the scenario of pulmonary nodules' characterizing and lung cancer screening, respectively. Two risk models were developed within the subgroups of malignant and benign pulmonary nodules in this study. By adding the CN9 result to the Mayo model indicators, it achieved a sensitivity of 41.3% and an AUC of 0.74 at a specificity of 91.3%. By adding the CN9 result to the Brock model indicators, it achieved a sensitivity of 47.7% and an AUC of 0.78 at a specificity of 91.3%. Both were improved compared with either the standalone Mayo or Brock model.

**Discussion:**

This multi-center prospective study indicates a panel of nine autoantibody markers that can help in the detection of lung cancer and the classification of pulmonary nodules in the Chinese population.

## 1 Introduction

Lung cancer currently has the highest incidence and mortality among all cancers in China (1, 2). It resulted in 870,982 new cases and 766,898 deaths in 2022 (3), with one of the lowest 5-year survival rates at 19.7% (4). The prognosis of lung cancer highly depends on the stage of diagnosis. The survival rate is as high as 59% for localized lung cancer, and it declines to 31.7% and 5.8% for regional and distant cancers (5). Patients with early lung cancer are usually asymptomatic; therefore, approximately 75% of patients are diagnosed at more advanced stages with limited treatment options and a poor outcome (6). Early diagnosis and treatment of lung cancer are critical to improve the overall survival as well as patients' quality of life. With low-dose CT screening, the National Lung Screening Trial (NLST) demonstrated a relative reduction of 20.0% in mortality from lung cancer, as well as a significant stage shift at diagnosis (7, 8). However, LDCT screening is also suffering from a high rate of false positive results and potential overdiagnosis (7, 9). There is an unmet need for early detection tools for lung cancer with the aid of CT.

Innovative molecular biomarkers could be one of the supplements for the early detection of lung cancer (10, 11). An alternative biomarker is the tumor-associated autoantibody (TAAb), which is produced by the immune system when it is triggered by abnormal antigens produced during tumorigenesis. TAAbs are produced early in tumorigenesis and have a biologically amplified signature. Therefore, they are more detectable in the early stages of cancer, sometimes years before clinical symptoms are developed, compared with their corresponding antigens. Their detection can be achieved with popularly adopted and affordable clinical technologies such as ELISA. They are released into the peripheral blood with typical half-lives of up to 30 days and are stable outside the body with limited degradation by proteases, which makes the test non-invasive, easy to access, and repeatable. All those features make TAAbs a promising biomarker for the early detection of cancers (12). Multiple studies have indicated that individual TAAbs or a panel of TAAbs can be used as serum biomarkers to detect lung cancer at an early stage or distinguish malignant tumors from benign pulmonary nodules (13–15). Two 7-TAAb panels have been widely reported on lung cancer. One was developed by the group Richardson JF in the United Kingdom and released by Oncimmune (Early CDT-Lung), including NY-ESO-1, p53, GBU4-5, CAGE, HuD, SOX2, and MAGE-A4 (16–18). It was mainly developed and validated in European and American populations, among which more than 90% of cases were either smokers or ever-smokers. Its clinical performance in non-smokers is unknown. Another panel includes SOX2, p53, GAGE7, PGP9.5, MAGE-A1, CAGE, and GBU4-5, which is mainly validated in the Chinese population and demonstrates variable performance on both sensitivity (25.42%−65.70%) and specificity (57.90%−91.75%) (19–22). It is still necessary to explore novel autoantibody combinations that are fit for Chinese and other populations, among which more than 50% of patients are non-smokers. This large-scale prospective study was carried out to further validate candidate autoantibodies and potential autoantibody combinations for early detection of lung cancer in the Chinese population.

## 2 Materials and methods

### 2.1 Subjects and samples

Patients were prospectively enrolled when visiting the three clinical centers (Zhongshan Hospital of Fudan University, Henan Provincial People's Hospital, and Shanghai Chest Hospital) for routine physical examination, follow-up, diagnosis, or treatment during August 2019 and December 2020, under an Institutional Review Board-approved protocol (NCT04216511) (23). This study was approved by the Ethics Committee at Zhongshan Hospital of Fudan University, Henan Provincial People's Hospital, and Shanghai Chest Hospital, and was conducted according to the principles of the Declaration of Helsinki.

**Figure 1:**
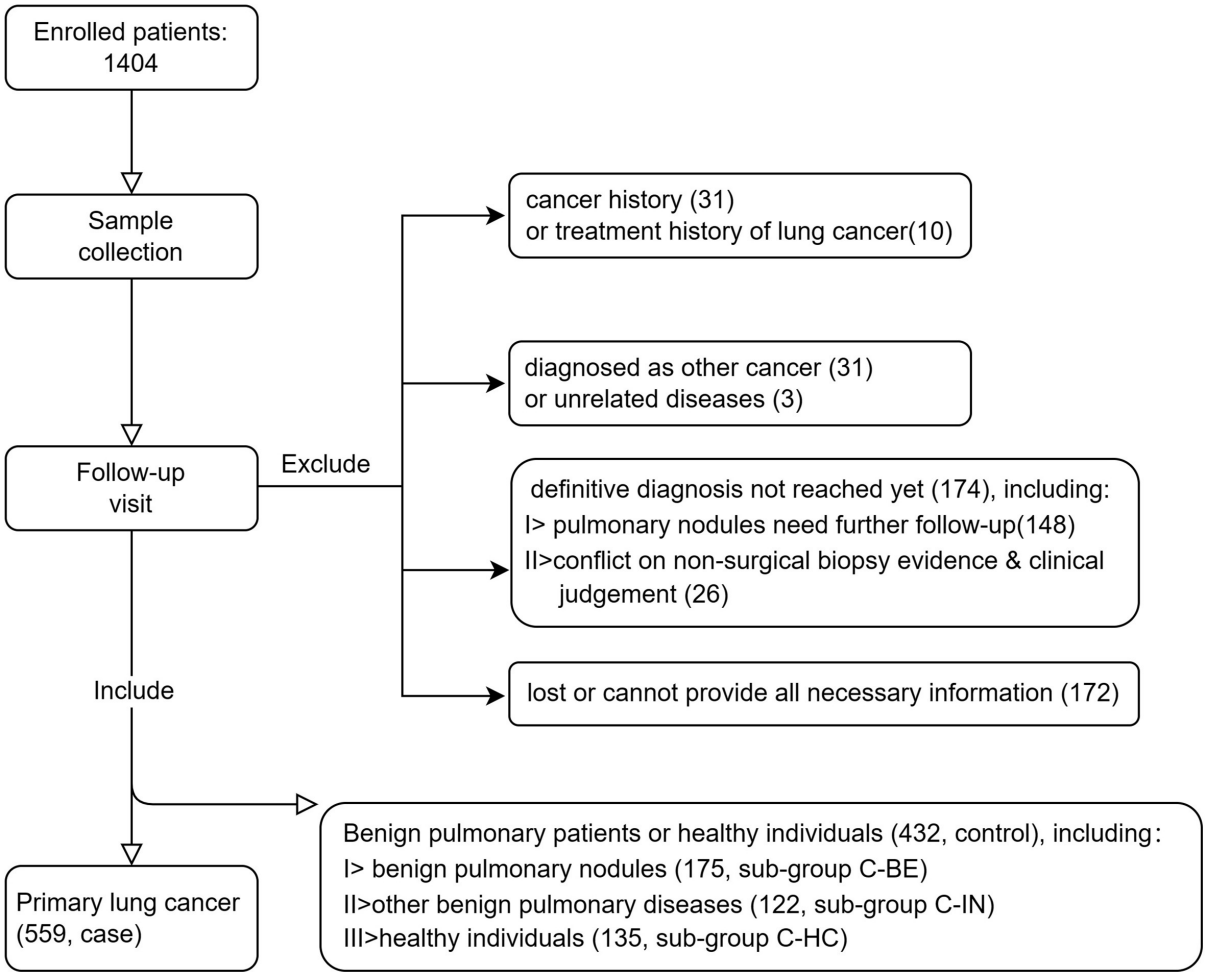
Schematic of patient inclusion.

The inclusion criteria are as follows: (1) male or female aged 18 years or above; (2) individuals complying with either of the following: pathologically diagnosed as lung cancer (case), or clinically diagnosed as benign pulmonary nodules (confirmed by pathology or by follow-up based on 2018 Chinese Consensus on Pulmonary Nodule Diagnosis and Treatment, control, subgroup C-BE) and other benign pulmonary diseases (e.g., COPD, infection, sarcoidosis, pulmonary fibrosis, etc., control, subgroup C-IN), or no pulmonary nodules or other obvious abnormalities were found in CT examination within 3 months before the enrollment (control, subgroup C-HC); and (3) the participant is willing and able to give informed consent for participation in the study, and provides the necessary information required. The exclusion criteria include (1) a history of any cancer other than basal cell carcinoma and (2) a lung cancer patient who has received or is receiving any treatment for cancer. In total, 1,404 patients were prospectively enrolled in the study. All participants signed the Informed Consent Form, filled out the survey, and had a chest CT scan. Peripheral blood (10 ml) specimens of each patient were collected in an EDTA tube by venipuncture. Consequently, 991 subjects who had a definite diagnosis were included in the development of the autoantibody panel. Patient inclusion details are summarized in Figure 1.

### 2.2 Quantification of autoantibodies in plasma

Plasma was separated from collected peripheral blood in an EDTA tube within 24 h by centrifugation (1,800 *g* for 10 min) and then stored at −80°C. All plasma samples were shipped to the central lab in the Zhongshan Hospital of Fudan University. With a quantitative ELISA assay, the concentrations of candidate autoantibodies were determined blindly. Corresponding antigens were provided by Oncimmune as described earlier, as were the protocol and analytical performance of the ELISA assay (23–25).

### 2.3 Statistics

Included cases and controls were randomly assigned to either the training or validation set, respectively. The performance of individual autoantibody candidates was evaluated first by its sensitivity at a specificity of 95% in the training set. Then, a composite panel of several autoantibodies was developed using a Monte-Carlo simulated annealing method to distinguish matched lung cancer cases from controls in the set. A receiver operating characteristic (ROC) curve was constructed using a Monte-Carlo search method (referring to the work of Healey, G.F. and his colleagues) within a case-control cohort, which enabled high- and low-specificity versions of the autoantibody panel to be determined (26). The cutoffs achieving the maximum Youden index with a specificity greater than or equal to 90% were determined as the optimal cutoffs. Positive diagnostic ratios (LR^+^) and negative diagnostic ratios (LR^−^) were also calculated. Finally, logistic regression analysis was used to develop risk models for pulmonary nodule stratification, taking the CN9 result and Mayo indicators, or the CN9 result and Brock cancer probability, as two independent risk factors within the subgroup of malignant and benign pulmonary nodules in this study.

## 3 Results

### 3.1 Demographic characteristics of the study population

A total of 991 subjects who had a definite diagnosis were included in the development of the autoantibody panel. In the case arm, 559 patients pathologically diagnosed as primary lung cancer were included, of which 290 (51.9%) at early stages (Stages 0, I, and II, or limited), while 262 (46.9%) at advanced stages (Stages III and IV, or extensive), 385 (68.9%) were diagnosed as adenocarcinoma, 82 (14.7%) squamous carcinoma, and 59 (10.6%) small cell lung cancer. In total, 432 individuals who were healthy subjects (without clinically significant abnormality on CT) or diagnosed with benign pulmonary diseases (including benign pulmonary nodules) were included in the control arm, which included 135 (31.3%) healthy individuals, 175 (40.5%) patients with benign pulmonary nodules, and 122 (28.2%) patients with other benign pulmonary diseases. The mean age of cases was 61 years (range, 30–88 years), including 316 (56.5%) male and 243 (43.5%) female patients. The mean age of controls was 56 years (range, 18–84 years), including 246 (56.9%) male and 186 (43.1%) female patients. Detailed demographic and clinical characteristics are presented in Table 1.

**Table 1:** Demographic and clinical characteristics of the study population.

	**Training set (*****n*** = **644)**					**Validation set (*****n*** = **347)**				
	**Case (*****n*** = **363)**		**Control (*****n*** = **281)**		***p*** **-value**	**Case (*****n*** = **196)**		**Control (*****n*** = **151)**		***p*** **-value**
Age (average, range)	61 (30–88)		56 (18–84)		<0.001	61 (31–87)		56 (18–84)		<0.001
**Gender**										
Male	203	55.92%	167	59.43%	0.37	113	57.65%	79	52.32%	0.32
Female	160	44.08%	114	40.57%		83	42.35%	72	47.68%	
**Smoking history**										
Non-smoker	141	38.84%	141	48.45%	<0.001	62	31.63%	70	46.36%	0.001
Current smoker										
- Smoking Index ≥ 400	60	16.53%	24	8.54%		41	20.92%	16	10.60%	
- Smoking Index < 400	16	4.41%	24	8.54%		8	4.08	4	2.65%	
Former smoker										
- Smoking Index ≥ 400 and quit < 15 year	43	11.85%	24	8.54%		24	12.24%	6	3.97%	
- Smoking Index < 400 or quit >15 year	101	27.82%	61	21.71%		60	30.61%	50	33.11%	
Unclear	2	0.55%	7	2.41%		1	0.51%	5	3.31%	
**Family history of lung cancer**										
First-degree relative	29	7.99%	25	8.90%	0.095	12	6.12%	6	3.97%	0.18
Any reported	3	0.83%	9	3.20%		0	0%	2	1.32%	
**Underlying lung disease**										
COPD	5	1.38%	21	7.47%	<0.001	5	2.55%	1	0.66%	0.058
Interstitial lung disease	3	0.83%	2	0.71%		0	0%	0	0%	
Tuberculosis	7	1.93%	4	1.42%		2	1.02%	4	2.65%	
**Exposure history**										
Asbestos	1	0.28%	1	0.36%	0.69	0	0%	0	0%	0.68
Braize	1	0.28%	1	0.36%		1	0.51%	0	0%	
Passive smoking	51	14.05%	50	17.79%		28	14.28%	22	14.57%	
**Histology type of lung cancer**										
Adenocarcinoma	248	68.32%	–	–		137	69.90%	–	–	
Squamous carcinoma	57	15.70%	–	–		25	12.76%	–	–	
SCLC	38	10.47%	–	–		21	10.71%	–	–	
Others	9	2.48%	–	–		4	2.04%	–	–	
Unidentified	10	2.75%	–	–		9	4.59%	–	–	
Unclear	1	0.28%	–	–		0	0.00%	–	–	
**NSCLC staging**										
0	3	0.83%	–	–		4	2.04%	–	–	
I	138	38.02%	–	–		82	41.84%	–	–	
II	23	6.34%	–	–		11	5.61%	–	–	
III	55	15.15%	–	–		29	14.80%	–	–	
IV	101	27.82%	–	–		47	23.98%	–	–	
Unclear	5	1.38%	–	–		2	1.02%	–	–	
**SCLC staging**										
Limited	17	4.68%	–	–		12	6.12%	–	–	
Extensive	21	5.79%	–	–		9	4.59%	–	–	

### 3.2 Evaluation of individual autoantibodies in the training set

**Figure 2:**
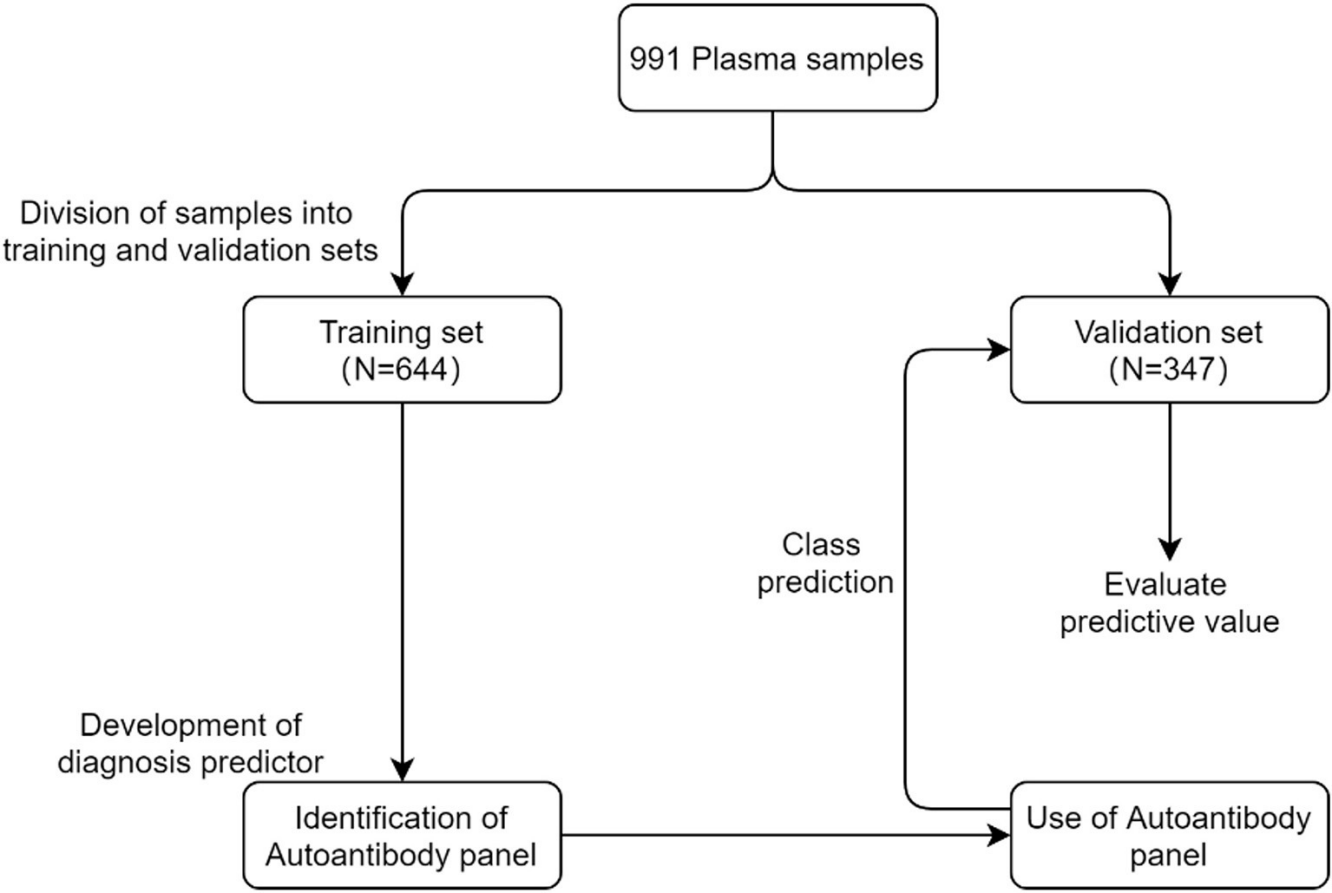
Overview of the strategy used for the development and validation of an autoantibody panel to identify lung cancer.

An overview of the strategy used for the development and validation of an autoantibody panel to identify lung cancer is shown in Figure 2. In total, 991 plasma samples were randomly divided into training and validation sets. The training set was first used to evaluate the performance of all tested autoantibody candidates individually, developing a diagnosis predictor, an autoantibody panel, and cutoffs. That autoantibody panel and cutoffs were then applied in the validation set to validate its clinical performance, as well as its sensitivity in subgroups of different stages, histological types, risk factors, and specificity in healthy controls, benign nodules, and other benign pulmonary diseases.

The training set consisted of 644 samples. Notably, 363 were pathologically confirmed cases, of which 49.9% were at the early stages (Stages 0, I, II, and limited) and 48.8% were at the advanced stages (Stages III, IV, and extensive). Histologically, 68.3% of the cases were adenocarcinoma, 15.7% squamous carcinoma, 10.5% small cell carcinoma, 0.6% large cell carcinoma, and 4.9% unidentified or other types. The control cohort included 281 plasma samples from healthy subjects (without clinically significant abnormalities on CT) or benign pulmonary diseases (including benign pulmonary nodules) (Table 1).

Fourteen autoantibody candidates, such as SOX2, P53, P53-95, SSX1, CK8, GBU4-5, HuD, CAGE, NY-ESO-1, α-enolase 1, MAGE-A4, KRAS, CK20, and P62, were measured in this study. The coefficient of variation of the ELISA assay was less than 10%, and the recovery was 90%−110%. The individual performance of the candidates was evaluated by its sensitivity at a specificity of 95% in the training set (Table 2). P53 and its isoform, P53-95, showed the highest sensitivities of 15.4% and 13.5%, respectively, among all the candidates, followed by SOX2 (12.4%), NY-ESO-1 (10.7%), and P62 (10.5%), whose sensitivities were more than 10%. Five candidates showed sensitivities between 10% and 6%, including CK8 (8.8%), HuD (8.5%), MAGE-A4 (6.9%), KRAS (6.9%), and CAGE (6.6%). The other four candidates showed sensitivities only slightly higher than 5%, including SSX1 (5.8%), α-enolase 1 (5.8%), GBU4-5 (5.5%), and CK20 (5.2%). Since the results of P53 and P53-95 were highly overlapped, only P53 was used for subsequent panel development.

**Table 2:** Individual performance of 14 autoantibody candidates in the training set.

	**P53**	**SOX2**	**CK8**	**SSX1**	**GBU4-5**	**P53-95**	**HuD**	**NY-ESO-1**	**CAGE**	**MAGE-A4**	**α-enolase-1**	**KRAS**	**P62**	**CK20**
Cutoff	2.22	3.22	2.37	3.16	3.71	3.01	2.65	16.08	2.71	5.21	6.06	6.06	10.55	5.0
Specificity	95.0%													
Sensitivity	15.4%	12.4%	8.8%	5.8%	5.5%	13.5%	8.5%	10.7%	6.6%	6.9%	5.8%	6.9%	10.5%	5.2%

### 3.3 Development of the autoantibody panel CN9 in the training set

**Figure 3:**
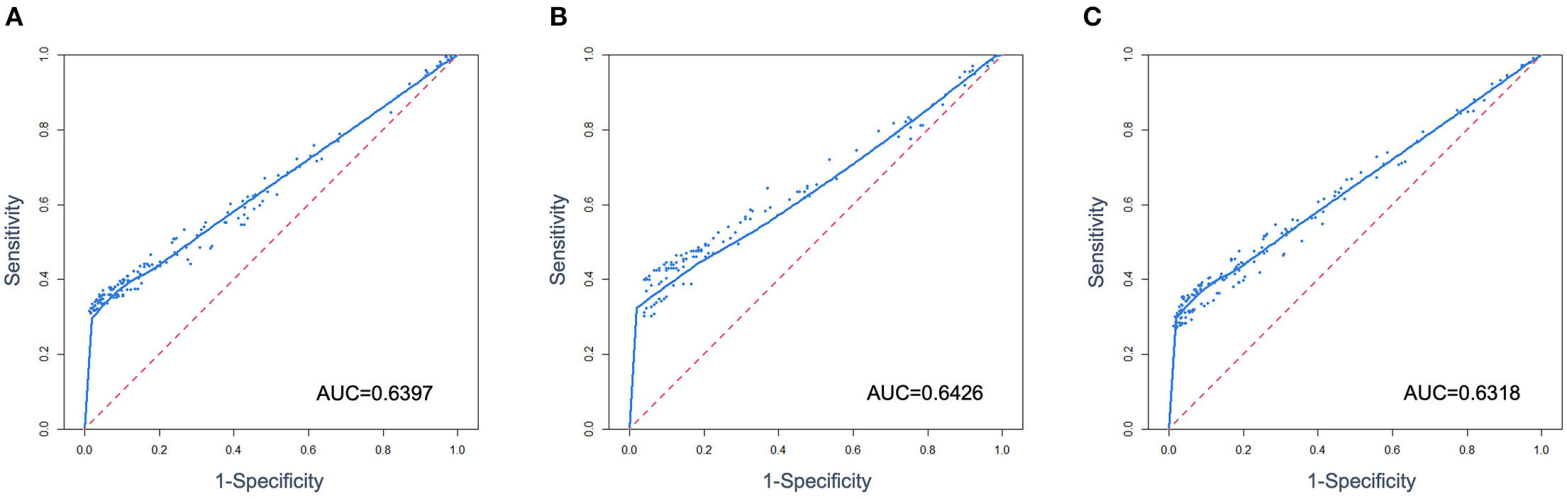
ROC curves were constructed from multivariate data from the training **(A)**, validation set **(B)**, and overall case-control cohort **(C)**.

A composite panel of nine autoantibodies (named CN9) was screened out to distinguish between cases and controls by the Monte-Carlo simulated annealing method, including P53, SOX2, SSX1, HuD, NY-ESO-1, CAGE, MAGE-A4, P62, and CK20. A ROC curve using a case-control cohort was constructed by the Monte-Carlo search method, and the area under the curve (AUC) was 0.6397 (Figure 3). A cutoff panel with a specificity ≥90% and the maximum Youden index was selected as the optimal cutoff panel to distinguish between cases and controls. Cutoffs for each autoantibody were 2.63 (P53), 7.89 (SOX2), 3.30 (SSX1), 4.78 (HuD), 25.89 (NY-ESO-1), 4.31 (CAGE), 7.30 (MAGE-A4), 13.20 (P62), and 7.00 (CK20), respectively, with the unit of U/ml for all the markers. CN9 showed a sensitivity of 41.3% (36.4%−46.5%, 95% CI) and a specificity of 90.0% (86.0%−93.0%, 95% CI) in the training set. The Youden index was 0.31, and the positive diagnostic ratio (LR+) and negative diagnostic ratio (LR-) were 4.15 and 0.65, respectively (Table 3).

**Table 3:** Clinical performance of CN9.

	**Specificity**	**95% CI**		**Sensitivity**	**95% CI**		**Youden Index**	**LR^+^**	**LR^−^**	**AUC**
Training set	90.04%	85.98%	93.02%	41.32%	36.37%	46.45%	0.31	4.15	0.65	0.6397
Validation set	91.39%	85.83%	94.90%	39.80%	33.20%	46.78%	0.31	4.62	0.66	0.6426
Overall	90.51%	87.38%	92.93%	40.79%	36.79%	44.91%	0.31	4.30	0.65	0.6318

### 3.4 Validation of CN9 in the validation set

The validation set consisted of 347 samples, of which 196 were pathologically confirmed cases and 151 were controls. A total of 55.6% of the cases were at the early stages (Stages 0, I, and II) and 43.4% at the advanced stages (Stage III and IV). A total of 69.9% of the cases were adenocarcinoma, 12.8% squamous carcinoma, 10.7% small cell carcinoma, 6.6% unidentified, or other types of lung cancer (Table 1). In total, 78 lung cancers and 13 controls were diagnosed as positive by CN9, resulting in a sensitivity of 39.8% (33.2%−46.8%, 95% CI) and a specificity of 91.4% (85.8%−94.9%, 95% CI) in the validation set. The Youden index was 0.31, and the positive diagnostic ratio (LR+) and negative diagnostic ratio (LR-) were 4.62 and 0.66, respectively (Table 3). The ROC curve showed an AUC of 0.6426 (Figure 3).

### 3.5 Overall clinical performance of CN9

The overall specificity and sensitivity of CN9 were 90.5% (87.4%−92.9%, 95% CI) and 40.8% (36.8%−44.9%, 95% CI). The Youden index was 0.31. The positive diagnostic ratio (LR+) and negative diagnostic ratio (LR-) were 4.30 and 0.65, respectively (Table 3). The AUC was 0.6318 (Figure 3). The overall accuracy was 85.5% and 90%, with PPV at 0.32 and 0.04, and NPV at 0.93 and 0.99 in the scenario of pulmonary nodules' characterizing and lung cancer screening, respectively, in terms of the estimated 10% malignant rate of pulmonary nodules in all non-calcified nodules >5 mm and 1% incidence of lung cancer in the population older than 40 years (27–34).

**Figure 4:**
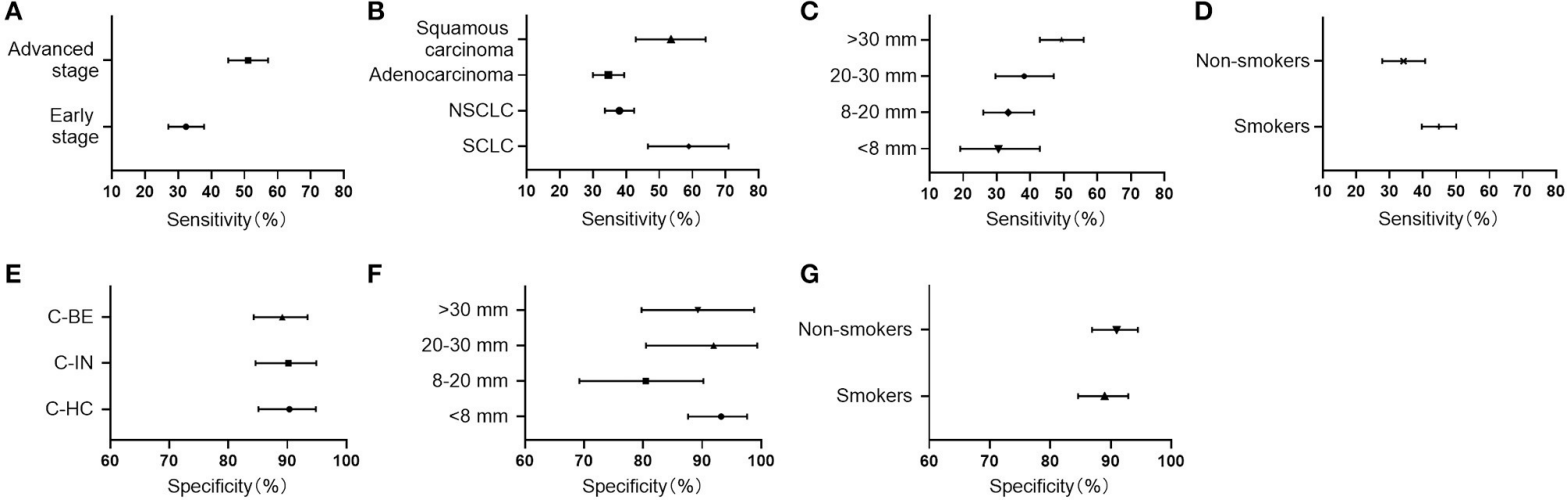
Clinical performance of CN9 in subgroups. **(A–D)** The sensitivities in subgroups of different stages, histological types, lesion sizes, and risk factors. **(E)** The specificities in healthy controls (C-HC), benign nodules (C-BE), as well as other benign pulmonary diseases (C-IN). **(F, G)** The specificities in subgroups of different lesion sizes and risk factors.

**Figure 5:**
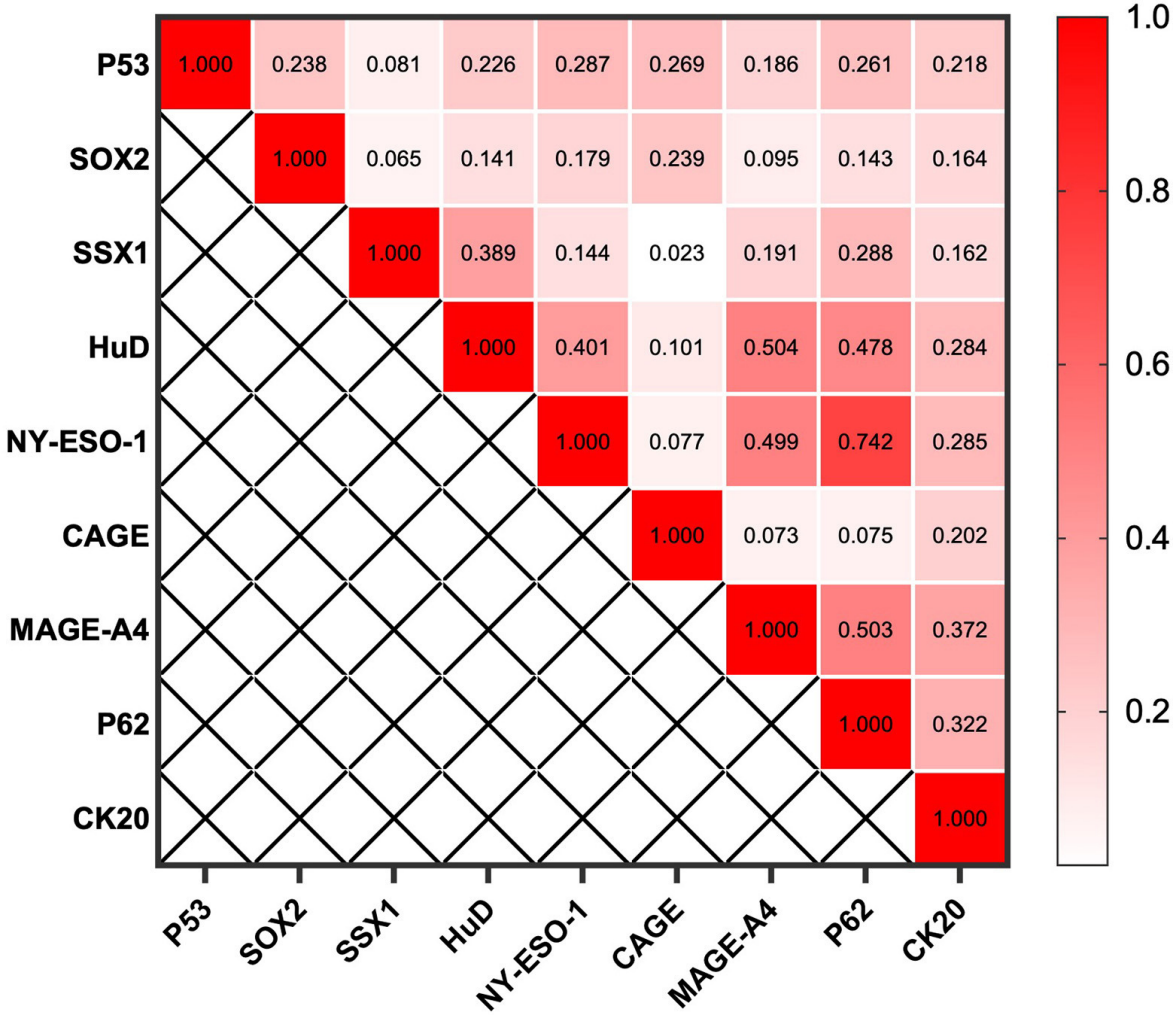
Spearman's rank correlation coefficient between individual autoantibodies of the CN9 panel.

CN9 showed a sensitivity of 32.2% (27.0%−37.8%, 95% CI) in early-stage lung cancer (Stages I and II, or limited), which is significantly lower than the sensitivity of 47.8% (41.5%−54.3%, 95% CI) in advanced-stage lung cancer (Stages III and IV, or extensive) (*P* = 0.0004) (Figure 4). The individual autoantibodies of CN9 revealed a similar trend (Table 4), although the majority of the antibodies showed a relatively low association except for NY-ESO-1 and p62 (Figure 5).

**Table 4:** The sensitivities of individual autoantibodies to CN9 in the early and advanced stages of lung cancer.

		**p53**	**SOX2**	**SSX1**	**HuD**	**NY-ESO-1**	**CAGE**	**MAGE-A4**	**p62**	**CK20**
Training set	Early stages	9.39%	6.08%	3.87%	4.97%	4.42%	2.76%	3.87%	4.97%	3.31%
	Advanced stages	19.77%	10.73%	7.34%	5.65%	7.91%	6.78%	3.39%	5.65%	3.39%
Validation set	Early stages	12.84%	5.50%	3.67%	1.83%	4.59%	3.67%	0.92%	3.67%	2.75%
	Advanced stages	21.18%	4.71%	14.12%	7.06%	8.24%	4.71%	7.06%	9.41%	4.71%
Overall	Early stages	10.69%	5.86%	3.79%	3.79%	4.48%	3.10%	2.76%	4.48%	3.10%
	Advanced stages	20.23%	8.78%	9.54%	6.11%	8.02%	6.11%	4.58%	6.87%	3.82%

The highest sensitivity of CN9 was shown in small cell lung cancer, which was 59.3% (46.6%−70.9%, 95% CI), followed by 53.7% (42.2%−64.0%, 95% CI) in squamous carcinoma, and 34.5% (30.0%−39.4%, 95% CI) in adenocarcinoma. The overall sensitivity in NSCLC was 37.9% (33.6%−42.4%, 95% CI) (Figure 4).

Considering the tumor size, CN9 demonstrated a positive rate of 29.6% (19.1%−42.8%, 95% CI), 33.1% (26.0%−41.1%, 95% CI), 37.9% (29.6%−47.0%, 95% CI), and 49.3% (42.8%−55.9%, 95% CI) in the lung cancer cases with lesion size <8 mm, 8–20 mm, 20–30 mm, and > 30 mm, respectively (Figure 4), with a specificity of 94.4% (87.6%−97.6%, 95% CI), 82.0% (69.2%−90.2%, 95% CI), 96.0% (80.5%−99.3%, 95% CI), and 91.9% (78.7%−97.2%, 95% CI), respectively (Figure 4). Diagnostic sensitivity in patients with tumors larger than 30 mm was significantly higher than that in patients with pulmonary nodules smaller than 30 mm, while the specificity remained high in patients with all lesion sizes.

CN9 showed a sensitivity of 44.8% (39.7%−50.0%, 95% CI) in smokers and ever-smokers, which was higher than that of 34.0% (27.8%−40.7%, 95% CI) (*P* = 0.005) in non-smokers (Figure 4). The diagnostic specificity of CN9 in smokers and ever-smokers was 89.5% (84.6%−92.9%, 95% CI), slightly lower than 91.5% (86.9%−94.5%, 95% CI) (*P* = 0.01) in non-smokers (Figure 4).

CN9 showed a specificity of approximately 90% in all three subtypes of controls in this study, including healthy individuals with matched gender and age, patients with other benign pulmonary conditions, and patients with benign pulmonary nodules, which were 91.1% (85.1%−94.8%, 95% CI), 91.0% (84.6%−94.9%, 95% CI), and 89.7% (84.3%−93.4%, 95% CI), respectively (Figure 4).

### 3.6 Combined risk model of pulmonary nodule stratification

**Figure 6:**
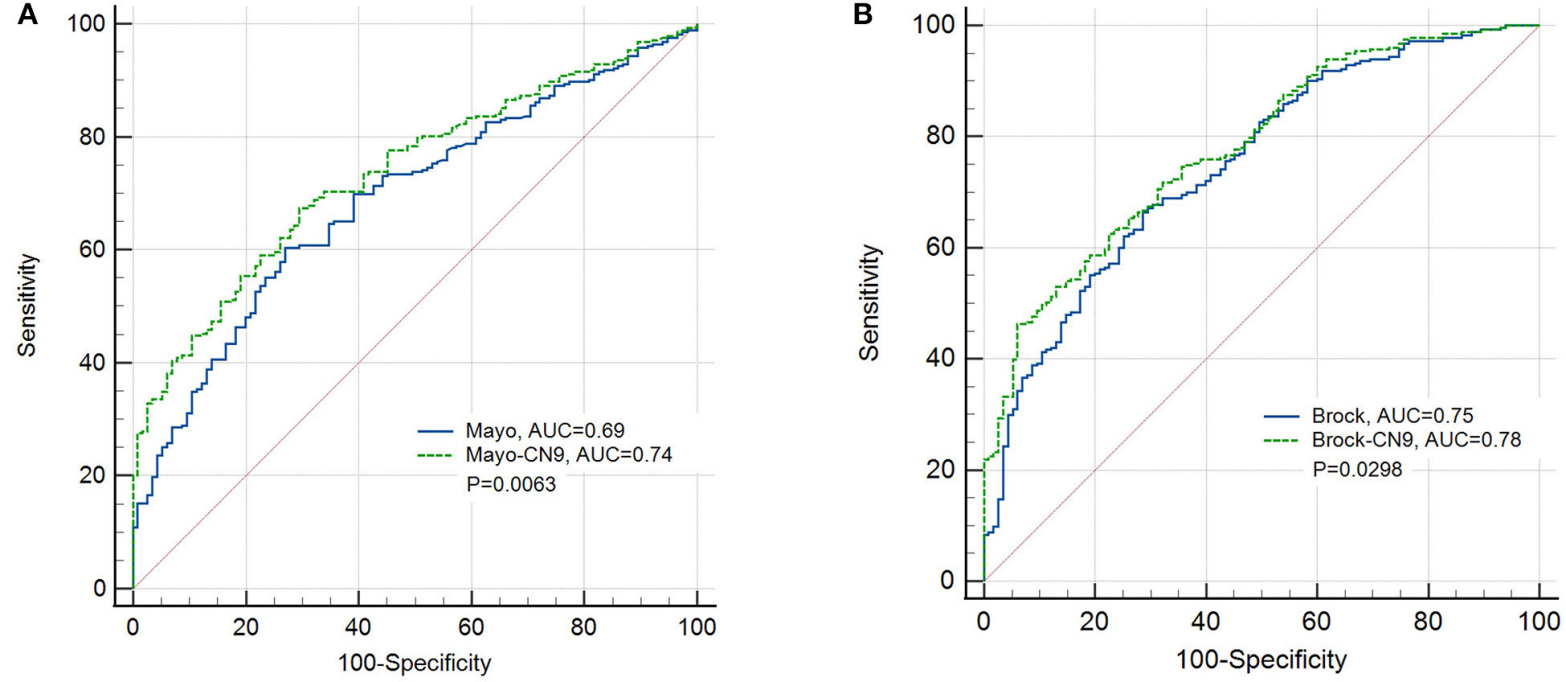
ROC curves of the prediction models of the Mayo Clinic and the newly developed Mayo-CN9 model **(A)**, the Brock and the newly developed Brock-CN9 model **(B)** in the pulmonary nodule subgroup.

There were 283 cases and 115 controls included in this study that could be identified as subjects with malignant or benign pulmonary nodules, and they had all the necessary information to calculate the risk indicator based on the prediction models of Mayo Clinic and Brock. In pulmonary nodule classification, the CN9 panel achieved a specificity of 91.3% (84.7%−95.2%, 95% CI), a sensitivity of 33.6% (28.3%−39.3%, 95% CI), and an AUC of 0.62 (0.584–0.665, 95% CI) in this subgroup. Mayo indicator and Brock model achieved sensitivities of 29.0% (23.8%−34.56%, 95% CI) and 38.9% (33.2%−44.8%, 95% CI) at the same specificity, and AUCs of 0.69 (0.642–0.735, 95% CI) and 0.75 (0.705–0.792, 95% CI), with the cutoffs of 0.46 and 26.12, respectively. The newly developed prediction model combining Mayo indicator and CN9 result (Mayo-CN9 model) was described by the following equation: *P* = 1/[1 + *e*[−(−0.132 + 1.539*CN9 *status* + 3.331*Mayo indicator)]], and the Brock-CN9 combined model was described as *P* = 1/[1 + *e*[–(–0.213 + 1.476*CN9 status + 0.059* Brock cancer probability)]], in which “P” is the probability of malignancy, “e” is the base of the natural logarithm, CN9 status equals to 1 if the detection result of autoantibody panel is positive, otherwise equals to 0. At the same specificity of 91.3%, the new Mayo-CN9 and Brock-CN9 models achieved sensitivities of 41.3% (35.5%−47.3%, 95% CI) and 47.7% (41.8%−53.7%, 95% CI) in this subgroup, with cutoffs of 0.84 and 0.824, respectively. The AUCs were 0.74 (0.689–0.778, 95% CI) and 0.78 (0.736–0.820). The combination of the CN9 panel significantly improved the diagnostic performance of the Mayo indicator (*P* = 0.0063) and the Brock model (*P* = 0.0298). The performance of the CN9, Mayo, and Mayo-CN9 indicators and the Brock and Brock-CN9 models in this study is listed in Table 5. Their ROCs are shown in Figure 6.

**Table 5:** Pulmonary nodule stratification performance of the CN9, Mayo, Mayo-CN9, Brock, and Brock-CN9 models.

	**CN9**	**Mayo**	**Mayo-CN9**	**Brock**	**Brock-CN9**
Cutoff	N.A.	0.45	0.84	26.12	0.824
Specificity	91.3% (84.6%, 95.8%)				
Sensitivity	33.6% (28.3%, 39.3%)	29.0% (23.8%, 34.6%)	41.3% (35.5%, 47.3%)	38.9% (33.2%, 44.8%)	47.7% (41.8%, 53.7%)
AUC	0.62 (0.584, 0.665)	0.69 (0.642, 0.735)	0.74 (0.689, 0.778)	0.75 (0.705, 0.792)	0.78 (0.736, 0.820)

N.A. = Not available.

## 4 Discussion

In this multi-center prospective study, the concentrations of 14 candidate autoantibody markers in lung cancer patients were detected and compared with those of healthy individuals and patients with other benign pulmonary conditions. A lung cancer-specific autoantibody panel including 9-TAAb markers (CN9) was screened out, and its clinical performance was validated in an independent validation set. The overall sensitivity of CN9 was 40.8%, the specificity was 90.5%, and the AUC was 0.6318. The overall accuracy was 85.5% and 90% in the scenarios of pulmonary nodules' characterizing and lung cancer screening, respectively.

The CN9 panel included not only p53, SOX2, HuD, NY-ESO-1, CAGE, and MAGE-A4, the members of EarlyCDT-Lung, which have been broadly studied and applied in the United States and European countries, but also rarely reported SSX1, p62, and CK20. Those new members contributed to the improvement of sensitivity in non-smokers, from 28.5% of six EarlyCDT-Lung members to 34.0% of the CN9 panel, compensated for the limitations of EarlyCDT-Lung members in the non-smoking population, and made the new CN9 panel perform better and more balanced in the Chinese population.

Although CN9 showed a reasonable positive rate in subgroups of lung cancers at all stages, histological types, and tumor sizes, it differed from most other studies. Its sensitivity for early stages and smaller lesions is significantly lower than that for late stages and larger lesions. It suggests that the concentration of autoantibodies in the peripheral blood of patients is still positively related to tumor burden, like other biomarkers. Its relatively low positive rate in adenocarcinoma compared with squamous carcinoma and small cell lung cancer is more likely due to the much higher ratio of early-stage cases included for adenocarcinoma instead of the difference between histological types.

Patients with a cancer history were usually excluded from the diagnostic studies of TAAb, which shrank the clinical value of TAAb panels to some extent. Among the excluded subjects, six patients who had a history of cancers other than lung cancer were diagnosed with primary lung cancer. The positive rate of CN9 in those patients was 50.0%, quite comparable with its sensitivity in the included lung cancer cases, and significantly higher than the positive rate in the limited cases of other cancer patients (6/25, 24%).

Evidence from this study showed the prediction model combined with the CN9 autoantibody panel and classical models, either Mayo or Brock, had better performance than any of the models standalone in pulmonary nodule classification, which implies the potential implementation of such a combination in the early diagnosis of lung cancer. However, it is still necessary to further validate either of the models in a large, independent cohort.

There are some limitations to this study. First, there is a significant difference in age and smoking history between the case and control arms. This is mainly due to the difficulty of including matched controls for each case in such a prospective study. The population visiting hospitals for benign disease or routine physical examination is generally younger and less smoker as well. To elucidate its possible influence, a subgroup of 336 pairs of age, gender, and smoking status-matched case-control was further analyzed, which revealed a highly consistent performance of CN9 with the overall study population (Supplementary Tables 1, 2, Figures 1, 2). Second, cancer history, as one of the important risk factors, is insufficient in the risk model development since subjects with a cancer history were excluded.

## 5 Conclusion

This multi-center prospective study indicates a panel of nine autoantibody markers (CN9) can help in the detection of lung cancer and the classification of pulmonary nodules, especially in the Chinese population.

## Data availability statement

The original contributions presented in the study are included in the article/Supplementary material, further inquiries can be directed to the corresponding author.

## Ethics statement

The studies involving humans were approved by Zhongshan Hospital of Fudan University, Henan Provincial People's Hospital and Shanghai Chest Hospital. The studies were conducted in accordance with the local legislation and institutional requirements. The participants provided their written informed consent to participate in this study.

## Author contributions

LT, DW, XH, HL, and CB contributed to the conceptualization of the study. LT, JS, XZ, DG, YL, and JZ contributed to the project administration, methodology, data curation, and formal analysis. CB contributed to the supervision. LT contributed to the writing—original draft. CB, JS, XZ, DG, YL, JZ, DW, XH, and HL contributed to the writing—review and editing. All authors read and approved the final manuscript.
