# Supplementary material for: Development of an autoantibody panel for early detection of lung cancer in the Chinese population
Source: Front Med (Lausanne). 2023 Nov 27;10:1209747. doi: 10.3389/fmed.2023.1209747 (PMC10711285; doi:10.3389/fmed.2023.1209747)
Supplement: Supplementary file 1 [file Table_1.DOCX]

**Supplementary Materials**

**Supplementary Table 1.** Demographic and clinical characteristics of age, gender and smoking status matched subgroup of case and control.

|  | Case (n=336) | | Control (n=336) | | p-value |
| --- | --- | --- | --- | --- | --- |
| Age (mean, SD) | 58.94 (11.38) | | 58.64(11.16) | | 0.725 |
| Gender： |  |  |  |  |  |
| Male | 186 | 55.4% | 186 | 55.4% | 1 |
| Female | 150 | 44.6% | 150 | 44.6% |  |
| Smoking history： |  |  |  |  |  |
| Non-smoker | 170 | 46.4% | 170 | 46.4% | 1 |
| Smoker | 180 | 53.6% | 180 | 53.6% |  |
| Histological type of lung cancer： |  |  |  |  |  |
| Adenocarcinoma | 244 | 72.6% |  |  |  |
| Squamous carcinoma | 39 | 11.6% |  |  |  |
| SCLC | 32 | 9.5% |  |  |  |
| Others | 10 | 3.0% |  |  |  |
| Unidentified | 10 | 3.0% |  |  |  |
| Unclear | 1 | 0.3% |  |  |  |
| NSCLC staging： |  |  |  |  |  |
| 0 | 3 | 0.9% |  |  |  |
| I | 142 | 42.2% |  |  |  |
| II | 16 | 4.8% |  |  |  |
| III | 52 | 15.5% |  |  |  |
| IV | 88 | 26.2% |  |  |  |
| Unclear | 3 | 0.9% |  |  |  |
| SCLC staging： |  |  |  |  |  |
| Limited | 15 | 4.5% |  |  |  |
| Extensive | 17 | 5.0% |  |  |  |

Supplementary Table 2. Clinical performance of CN9 in matched and overall study population.

|  | Specificity | 95% CI | | Sensitivity | 95% CI | | Youden Index | LR^+^ | LR^-^ | AUC |
| --- | --- | --- | --- | --- | --- | --- | --- | --- | --- | --- |
| Matched | 91.4% | 87.9% | 93.9% | 40.2% | 35.1% | 45.5% | 0.32 | 4.67 | 0.65 | 0.622 |
| Overall | 90.5% | 87.4% | 92.9% | 40.8% | 36.8% | 44.9% | 0.31 | 4.30 | 0.62 | 0.632 |


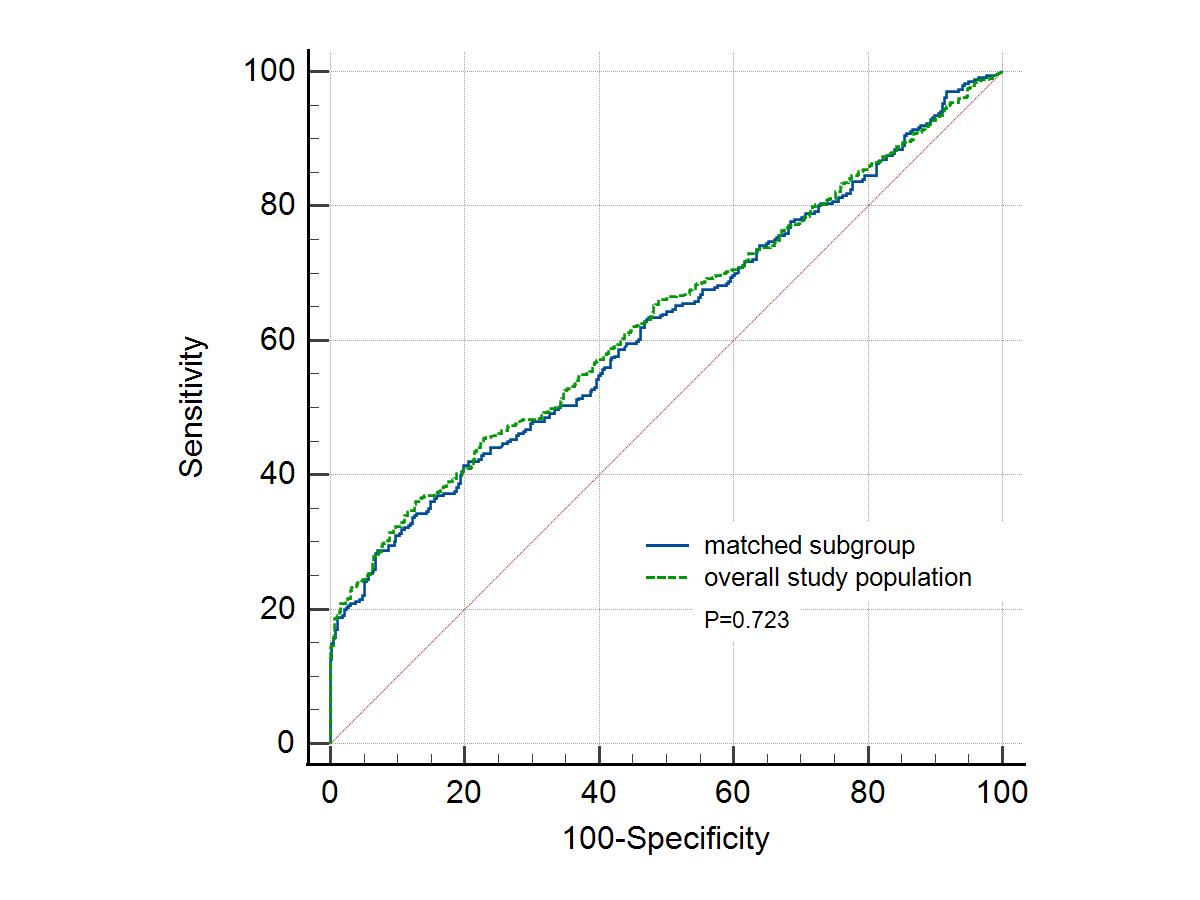


Supplementary Figure 1. ROC curves of CN9 panel in matched and overall study population.


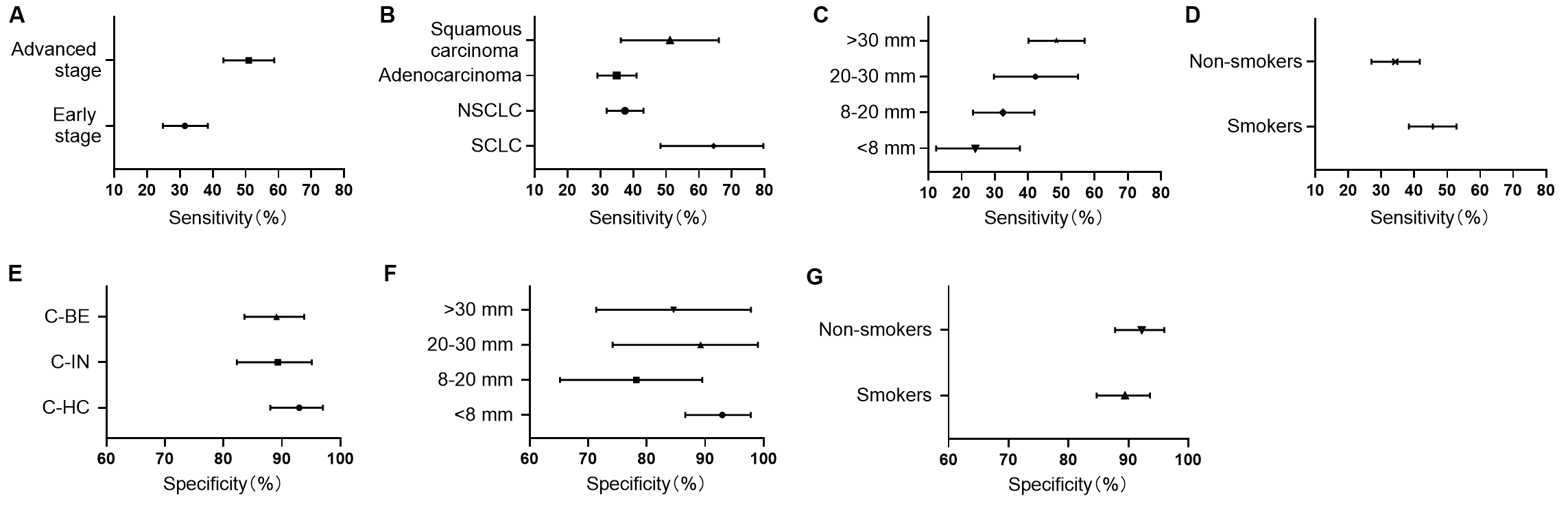


**Supplementary Figure 2.** Detailed clinical performance of CN9 in matched subgroup. A-D: The sensitivities in matched subgroup stratified by stages, histological types, lesion sizes and risk factors. E: The specificities in healthy controls (C-HC), benign nodules (C-BE) as well as other benign pulmonary diseases (C-IN) of the matched subgroup. F and G: The specificities in matched subgroup stratified by lesion sizes and risk factors.
